# Decongestion in patients with advanced chronic kidney disease coexisting with heart failure

**DOI:** 10.1093/ckj/sfaf185

**Published:** 2025-06-12

**Authors:** Carmine Zoccali, Adeera Levin, Francesca Mallamaci, Robert Giugliano, Raffaele De Caterina

**Affiliations:** Renal Research Institute, New York, NY, USA; Institute of Molecular Biology and Genetics (Biogem), Ariano Irpino, Italy; Associazione Ipertensione Nefrologia Trapianto Renal (IPNET), c/o Nefrologia, Grande Ospedale Metropolitano, Reggio Calabria, Italy; Division of Nephrology, Department of Medicine, University of British Columbia, Vancouver, BC, Canada; Associazione Ipertensione Nefrologia Trapianto Renal (IPNET), c/o Nefrologia, Grande Ospedale Metropolitano, Reggio Calabria, Italy; Cardiovascular Division, Brigham and Women's Hospital, Harvard Medical School, Boston, MA, USA; Chair of Cardiology, University of Pisa and Cardiology Division, Pisa University Hospital, Pisa, Italy

**Keywords:** brain natriuretic peptide, CKD, heart failure, pulmonary congestion, SGLT-2 inhibitors

## Abstract

Heart failure (HF) and chronic kidney disease (CKD) are closely interconnected conditions. Congestion, a central element in HF and CKD pathophysiology, progresses from haemodynamic changes to pulmonary oedema, with asymptomatic pulmonary congestion and an isolated increase in brain natriuretic peptide (BNP) as an intermediate step. Management strategies include sodium restriction, diuretics and emerging technologies for fluid monitoring. Diuretics, while essential, present challenges such as resistance and side effects, necessitating combination therapies and alternatives, like SGLT-2 inhibitors and, in special cases, ultrafiltration. Personalized approaches are critical to improving clinical outcomes in HF and CKD.

Congestion is a cardinal manifestation of heart failure (HF) [1]. The events leading to pulmonary oedema during heart failure can be conceptualized as a cascade [2], with clinical pulmonary congestion being a late event that occurs days or weeks after exposure to high left ventricular end-diastolic pressure and pulmonary capillary wedge pressure (haemodynamic congestion). The imbalance of Starling's equilibrium at the alveolar-capillary level (Fig. 1) is central to the clinical emergence of congestion [3]. Between haemodynamic and clinical pulmonary congestion (Fig. 2), an intermediate stage in the presymptomatic phase, detectable at lung auscultation or more often by lung ultrasound (US) (US-B lines) of chest X-ray Kerley B lines (Fig. 1) or an increase in levels of brain natriuretic peptide (BNP), which may act as indicators and intermediate endpoints of frank pulmonary oedema. A total of 32% of HF patients seen in outpatient settings display evidence of excessive lung water by US studies and 81% have no findings on auscultation [4].

**Figure 1:**
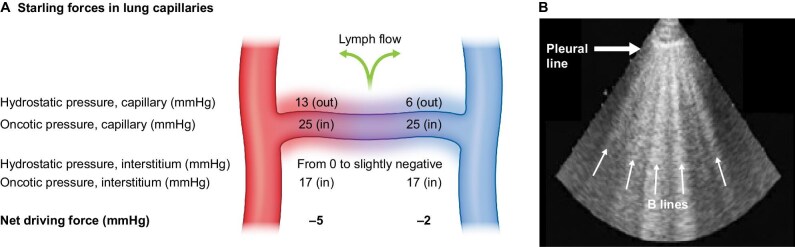
Starling forces at the capillary level in the lung. The lung is a unique territory where hydrostatic pressure is peculiarly regulated. Net filtration pressure at the alveolar level is zero or negative, which protects the lung from the risk of pulmonary oedema in volume expansion states like CKD and HF. A constant flow of lymphatic fluid from the pulmonary interstitium to the systemic circulation guarantees the protective lung microhaemodynamic setting. Moreover, the lung has a uniquely high interstitial compliance, like in nephrotic syndrome, characterized by low oncotic pressure. In conditions of excess extravascular lung water, the subpleural interlobular septa thickened by oedema reflect the ultrasound (US) beam. This phenomenon generates reverberation artifacts (‘comet tails’) called B-lines, the US equivalent of Kerley B-lines on a chest X-ray.

**Figure 2:**
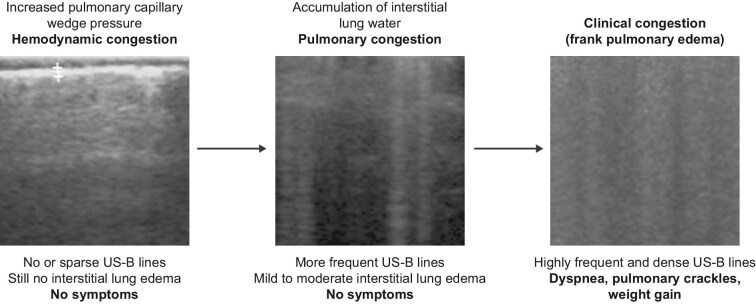
The imbalance of Starling's equilibrium at the alveolar-capillary level is central to generating congestion. Between haemodynamic and clinical pulmonary congestion, the intermediate event in the presymptomatic phase is an imaging sign detectable by lung ultrasound (US) as a US equivalent (US B-lines) to chest X-ray B-lines (Fig. 1). This sign may act as an indicator and an intermediate endpoint of frank pulmonary oedema.

The risk of congestion is magnified in chronic kidney disease (CKD) patients, particularly in stage 5D CKD patients. Intermittent fluid removal during haemodialysis (HD) sessions results in fluid accumulation and rapid depletion [5], translating into a cyclical haemodynamic stress. Volume overload [6], a trigger and proxy of lung congestion, or congestion directly measured by lung US [7], are the strongest predictors of mortality in dialysis patients, surpassing traditional risk factors such as hypertension and diabetes.

Strategies to counter congestion in patients with HF and CKD include implementing dietary sodium restriction to minimize fluid retention, judiciously using diuretics in non-dialysis CKD patients and optimizing dialysis prescriptions to achieve adequate fluid removal in dialysis patients. Emerging technologies such as continuous monitoring of fluid status and personalized dialysis regimens hold promise for improving outcomes.

## SODIUM RESTRICTION

Recent studies have increasingly challenged the long-held belief in the benefits of severe dietary sodium and fluid restrictions for patients with HF [8]. These studies suggest that such stringent measures may not yield the expected improvements in clinical outcomes and could potentially lead to unintended negative effects. The European Society of Cardiology (ESC) HF guidelines reflect this evolving understanding by recommending sodium intake be limited to ≤2 g/day and fluid intake to 1.5–2 l/day, but only for selected patients with HF [9]. This selective approach acknowledges that while some patients may benefit from these restrictions, others may not, and overly strict limitations could exacerbate issues such as dehydration or electrolyte imbalances.

Similarly, the 2024 KDIGO guidelines for CKD advocate for sodium intake of <2 g/day [10]. This recommendation is crucial for controlling hypertension, a major risk factor for both the development and progression of HF. However, implementing such low sodium levels in everyday clinical practice poses significant challenges. Many patients struggle to adhere to these guidelines due to the high sodium content in processed and restaurant foods, as well as ingrained dietary habits [11].

The Chronic Renal Insufficiency Cohort study provides valuable insights into this issue. By using 24-h urine collections to estimate sodium intake, the study offers a precise measure of actual consumption. It found that the correlation between sodium intake and cardiovascular events levelled off at ≈130–150 mmol of sodium per day, which corresponds to ≈3 g of sodium daily [12]. This suggests that sodium intake at this level might serve as a more realistic and less restrictive target for patients with both HF and CKD. Such a target could help manage hypertension and reduce cardiovascular risk without imposing the difficulties associated with more severe restrictions. Adopting this more moderate approach could enhance patient compliance and improve clinical outcomes by setting dietary goals that are both effective and achievable. It underscores the importance of tailoring dietary recommendations to individual patient needs and circumstances rather than adhering to a one-size-fits-all model. This shift towards personalized care in managing HF and CKD could lead to better health outcomes and quality of life for patients.

As to fluid restriction, a multicentre open-label trial by Hermann *et al.* [13] explored the effects of liberal fluid intake versus fluid restriction (up to 1500 ml/day) in outpatients with chronic heart failure. The primary outcome, health status at 3 months assessed using the Kansas City Cardiomyopathy Questionnaire Overall Summary Score (KCCQ-OSS), showed no significant difference between the liberal intake group (74.0) and the fluid restriction group (72.2), with an adjusted mean difference of 2.17 [95% confidence interval (CI) −0.06–4.39; *P* = .06]. Secondary outcomes revealed higher thirst distress in the fluid restriction group, while safety events were similar across both groups. These results suggest that fluid restriction may not provide significant benefits in managing chronic heart failure, challenging its routine recommendation.

## DIURETICS

Loop diuretics are the preferred diuretic class in patients with coexisting HF and CKD and the cornerstone of management in HF, both in the acute and the chronic setting of this condition [14].

### Acute decompensated HF

**Figure 3:**
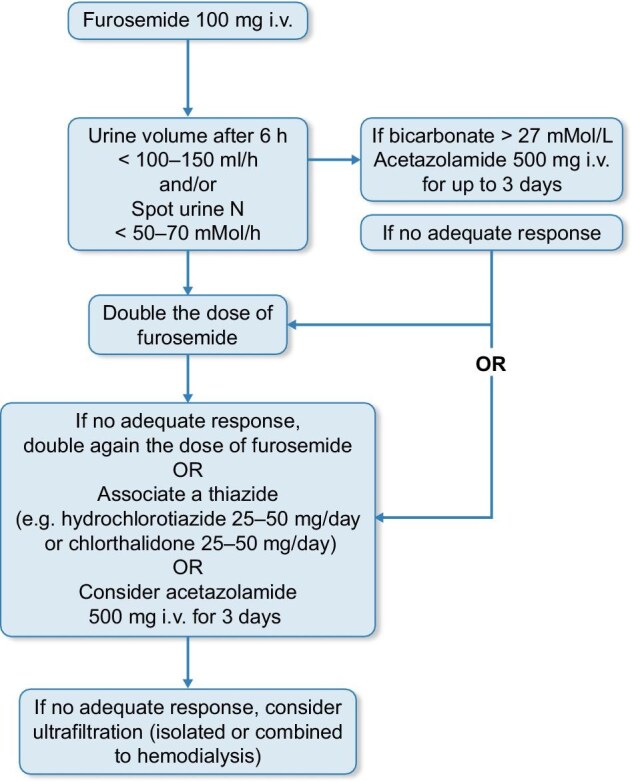
An algorithm for the use of diuretics in the treatment of acute decompensated HF. The first step is to administer intravenous or oral furosemide to diuretic-untreated or oral furosemide–treated patients. If this intervention fails to adequately increase urine volume and urinary sodium in patients with serum bicarbonate >27 mmol/l, the next step is to administer acetazolamide. If patients fail to respond to acetazolamide, either doubling the dose of furosemide or adding a thiazide is recommended. In unresponsive patients with bicarbonate <27 mmol/l, further doubling the furosemide dose or using thiazides or acetazolamide are possible options. Independent of serum bicarbonate, add a SGLT2i (see Table 1) in unresponsive patients. Finally, ultrafiltration, either isolated or associated with HD, can be considered in resistant patients.

The timely use of diuretics is lifesaving in acute HF. Fig. 3, simplified and adapted from an ESC consensus document [15], presents an algorithm for tailoring furosemide and other diuretics in this setting. Thiazide diuretics, once deemed ineffective in stage ≥4 CKD, have shown promise in advanced CKD [16] through sequential nephron target inhibition, now with well-documented efficacy [17]. The addition of thiazides to loop diuretics may worsen kidney function, however, kidney tubular injury does not appear to have an association with worsening kidney function in the context of aggressive diuresis in patients with acute HF [18].

Carbonic anhydrase inhibitors, in combination with loop diuretics, may also overcome diuretic resistance by blocking the proximal tubule compensatory response to loop diuretics. The addition of acetazolamide (500 mg/day intravenously for 3 days) to loop diuretic therapy resulted in a greater incidence of successful decongestion (42%) compared with placebo (31%) [19] and appears more effective in patients with an estimated glomerular filtration rate (eGFR) ≤40 ml/min/1.73 m^2^. Acetazolamide is safe and reduces hospital stay length but not rehospitalization rates or mortality. The drug is particularly effective in patients with elevated bicarbonate levels, as it counteracts a key component of diuretic resistance.

Mineralocorticoid receptor inhibitors (MRAs) are widely used alongside loop diuretics for secondary prevention of congestion in HF. Practical information about the use of these drugs in patients with kidney dysfunction is given in Table 1. High-dose (100 mg/day) and low-dose spironolactone (25 mg/day) are equally effective in reducing clinical congestion scores, dyspnoea metrics, net urine output, net weight change and 30-day mortality. Eplerenone and finerenone are similarly effective in reducing congestion, as estimated by N-terminal pro-brain natriuretic peptide (NT-proBNP) levels in patients with worsening renal function in chronic HF and diabetes with or without CKD [20]. An advantage of these medications is that they have a lower risk for hyperkalaemia compared with spironolactone, with finerenone being the drug with the lowest risk in this class for this side effect [21]. Finerenone improved the risk for worsening HF and death from cardiovascular causes in patients with mildly reduced or preserved ejection fraction (EF) [22]. However, in contrast with eplerenone [23], the use of finerenone in decompensated HF with reduced EF still needs proper validation in specific randomized trials. There is no labelled indication for the use of finerenone in the treatment of HF.

**Table 1:** Essential pharmacokinetic and clinical information about the use of oral diuretics, MRAs and SGLT2is in adults.

Drug	Oral bioavailability (%)	Half-life (hours)	Duration of action (hours)	Typical dose (mg/day)	Route of elimination and dose adjustment according to the eGFR (ml/min/1.73 m^2^)	Contraindication depending on the eGFR (ml/min/1.73 m^2^)
Diuretics						
Furosemide	47–64	0.5–2.0	6–8	20–80 (maximum 500)	50% K 50% faeces (unchanged)	Anuria
					>30 no dose adjustment	
					<30: up to 500 mg	
Bumetanide	59–89	1.0–1.5	4–6	0.5–2 (maximum 10)	81% K, 19% M	Anuria
					>30 no dose adjustment	
					<30 up to 10 mg	
Hydrochlorothiazide	65–75	6–15	6–12	25–50 (maximum 100)	≈100% K	<10
					>10 no dose adjustment	
					<10 ineffective	
Chlorthalidone	100	40–60	24–48	25–50 (maximum 100)	≈100% K	<10
					>10 no dose adjustment	
					<10 ineffective	
Metolazone	≈65–80	6–8	Up to 24	2.5–5 (maximum 20)	≈90% K, 10% M	<10
					No dose adjustment	
MRAs						
Spironolactone	≈65	≈1.6	48–72	25–50 (maximum 200)	M	<30
Eplerenone	69	≈5	≈48	25 (maximum 50)	M	<30
Finerenone	44		≈48	20 (maximum 20)	M	<30
SGLT2is						
Empagliflozin	78	12.4	≈72	10 (maximum 25)	50% M, 50% K	<20
					>45 no dose adjustment	
Dapagliflozin	78	≈12.9	≈72	5 (maximum 10)	21% M, 79% K	<30
					>45 no dose adjustment	
Canagliflozin	65	10–13	≈24	100 (max 300)	41% M, <1% K	<30
					>60 no dose adjustment	
Ertugliflozin	100	11–17	8–14	20 mg in four doses	98.5% M, 1.5% K	<25
					No dose adjustment	

In the ‘Route of elimination’ column, M refers to metabolic and K refers to kidney elimination.

Sodium–glucose co-transporter 2 inhibitors (SGLT2is), such as empagliflozin and dapagliflozin (Table 1), act on the nephron proximal tubules to induce glycosuria and osmotic diuresis. These drugs have shown significant benefits in both chronic and acute HF and are currently indicated for primary and secondary prevention of congestion. SGLT2is decrease intravascular volume, ventricular preload and myocardial oxygen consumption by reducing glucose and sodium reabsorption. They also promote a metabolic shift toward ketone utilization, enhancing myocardial energy efficiency. Early initiation of empagliflozin in acute HF patients is safe and increases urine output without significantly affecting natriuresis [24, 25]. Dapagliflozin improves natriuresis and urine output, reduces the time to intravenous diuretic discontinuation and shortens hospital stays [26]. Dapagliflozin and metolazone are equally effective in relieving congestion in HF patients with loop diuretic resistance [27]. These drugs are not necessarily alternatives and may be combined to relieve congestion. SGLT2is are generally not recommended for initiation in patients with an eGFR <20 ml/min/1.73 m^2^, and there is still no trial testing these drugs in dialysis patients.

Vasopressin antagonists, such as tolvaptan, block the action of antidiuretic hormone at vasopressin receptor type 2, promoting the excretion of free water. This drug increases fluid and weight loss in acute HF patients but increases the risk of worsening kidney function [28]. Combining tolvaptan with furosemide enhances diuretic efficacy to a degree comparable to that of metolazone and hydrochlorothiazide [29]. The American Heart Association (AHA)/American College of Cardiology (ACC)/Heart Failure Society of America (HFSA) [14] and ESC [30] guidelines recommend considering tolvaptan only in HF patients with congestion and hyponatremia. Overall, the use of tolvaptan is controversial due to the lack of consistent evidence in clinical trials, on long-term outcomes and its efficacy in all HF patients. While some studies show tolvaptan improves short-term congestion and symptoms, others lack conclusive evidence of long-term benefits in mortality or rehospitalization rates, worsening renal function despite its benefits in fluid and weight loss [31].

Ultrafiltration is a technique that extracts isotonic plasma water through a semipermeable membrane driven by a pressure gradient. The process requires the insertion of a central venous or large-lumen peripheral venous catheter and the use of anticoagulation to prevent clot formation. Unlike diuretics, ultrafiltration provides more predictable and controlled fluid removal without causing significant electrolyte imbalances or haemodynamic instability. Potentially it can be beneficial in patients who fail to respond adequately to high-dose diuretics. However, in the Cardiorenal Rescue Study in Acute Decompensated Heart Failure (CARRESS-HF) trial [32], ultrafiltration was inferior to pharmacologic therapy regarding the combined endpoint of serum creatinine level and body weight change 96 h post-enrolment, primarily due to an increase in creatinine levels in the ultrafiltration group. Specifically, the mean change in creatinine was −0.04 ± 0.53 mg/dl in the pharmacologic group compared with 0.23 ± 0.70 mg/dl in the ultrafiltration group (*P* = .003). There was no significant difference in weight loss between the groups, with the pharmacologic group losing 5.5 ± 5.1 kg and the ultrafiltration group losing 5.7 ± 3.9 kg (*P* = .58). Additionally, a higher percentage of patients in the ultrafiltration group experienced serious adverse events compared with the pharmacologic group (72% versus 57%; *P* = .03). Furthermore, ultrafiltration [32] was associated with a higher incidence of serious adverse events, including catheter-related complications and bleeding. The ESC [30], but not the AHA/ACC/HFSA guidelines [14], cautiously recommend ultrafiltration for diuretic-resistant patients with volume overload, emphasizing its role as a targeted therapy in carefully selected cases. However, ultrafiltration remains a resource-intensive intervention that requires specialized equipment and trained personnel, which restricts its availability to specific healthcare settings. Continuous venovenous hemofiltration can be potentially useful in critically ill patients with acute renal failure who cannot tolerate HD, although its use in HF remains controversial [33]. However, due to the lack of proper clinical trials, this option is not recommended in current guidelines [14, 30]. Hypertonic saline (3%) increases diuretic efficiency and improves metabolic derangements without adverse respiratory or neurologic signals [34]. However, its routine use is not recommended due to limited data and potential risks of sodium overcorrection. Additional studies of hypertonic saline as a diuretic adjuvant are warranted.

### Chronic HF

Loop diuretics are the preferred class of diuretics in patients with chronic HF and CKD [35]. Thiazides have long been considered of little efficacy in stage G4 or more severe CKD stages. However, a systematic review showed that these drugs may be helpful even among people with advanced CKD [36].

Appropriate use of diuretics in concert with other drugs is of central relevance in patients with coexisting HF and CKD [37]. In the 2021 ESC guidelines [30], diuretics and other guideline-directed medical therapies are recommended in patients with HF with reduced EF who have signs and/or symptoms of congestion to alleviate HF symptoms, improve exercise capacity and reduce HF hospitalizations. However, in the chronic HF setting, the benefits of diuretics, notably loop diuretics, in HF with preserved EF remain debated [38]. Along with the ESC and AHA/ACC/HFSA guidelines, information reported in Table 1 may guide the use of MRAs and SGLT2is in patients with chronic HF.

### Decongestion monitoring

Various strategies for fluid management in HF patients, including those with concomitant CKD, have been explored [39, 40]. In a meta-analysis of trials testing whether natriuretic peptide–guided treatment of HF reduces all-cause mortality and HF admissions in patients with HF (19 trials with 4554 participants), the risk ratio for all-cause mortality was 0.87 (95% CI 0.77–0.99) and that for HF admissions was 0.80 (95% CI 0.72–0.89). Sensitivity analyses, restricted to studies with a low risk of bias, produced similar estimates but were no longer statistically significant, indicating insufficient high-quality evidence to make definitive recommendations on the use of BNP/NT-proBNP-guided treatment in clinical practice [41]. Despite mixed results, BNP-guided therapy holds potential and future well-designed trials are needed to clarify its role in HF management.

As for lung US, a meta-analysis including 10 trials and 1203 patients showed that treatment strategies of acute decompensated HF guided by lung US reduce the risk for major adverse cardiovascular events (MACE) in comparison with standard clinical monitoring [relative risk (RR) 0.59 (95% CI 0.48–0.71)] as well as the rate of HF-related rehospitalization [RR 0.63 (95% CI 0.40–0.99)]. In this meta-analysis, the risks of attrition and reporting biases were low. However, no sensitivity analysis centred on trials with a low risk for bias was performed [42]. In patients on long-term HD, a treatment strategy guided by lung US, while failing to improve the primary endpoint (a composite of death and MACE) [43], showed a significant benefit for prevention of hospitalizations driven by acute, decompensated HF in a post hoc analysis [44]. Similar results were registered in another trial focusing on patients at low risk of congestion [45]. The ESC guidelines for HF, but not the AHA/ACC/HFSA guidelines [14], suggest lung US, along with NT-proBNP, for assessing pulmonary congestion.

Patient-reported outcomes, such as quality of life and symptom relief, are crucial in evaluating treatment efficacy and guiding decongestion strategies in HF and these outcome measures should be more systematically incorporated in future clinical studies.

In conclusion, the interplay between HF and CKD amplifies the complexity of treatment in patients with these coexisting conditions. This demands careful consideration of strategies such as dietary sodium restriction, judicious use of diuretics and ultrafiltration. Emerging therapies, including SGLT2 and carbonic anhydrase inhibitors, appear useful in diuretic-resistant cases. Lung US and biomarkers like NT-proBNP can detect early pulmonary congestion and be applied for treatment monitoring. While guidelines provide a framework for treatment, the heterogeneity of HF and CKD populations underscores the importance of individualized care. Future research should focus on refining these strategies, integrating novel technologies and addressing long-term management challenges to optimize clinical outcomes and quality of life for this high-risk patient group.

## Data Availability

No new data were generated for this review.
