# Correction to: Decongestion in patients with advanced chronic kidney disease coexisting with heart failure

**DOI:** 10.1093/ckj/sfaf288

**Published:** 2025-09-16

This is a **correction** to:

Carmine Zoccali, Adeera Levin, Francesca Mallamaci, Robert Giugliano, Raffaele De Caterina, Decongestion in patients with advanced chronic kidney disease coexisting with heart failure, *Clinical Kidney Journal*, Volume 18, Issue 7, July 2025, sfaf185, https://doi.org/10.1093/ckj/sfaf185

In the originally published version of this manuscript, there was an error on page 3, in the sentence quoting reference 12: excretion of 130-150 mmol of sodium per day does not correspond to intake of 3 grams of sodium chloride, instead it corresponds to intake of 3 grams of sodium.

This error has been corrected.

